# Relationship between the Metabolic Associated Fatty Liver Disease and Endometrial Thickness in Postmenopausal Women: A Cross-sectional Study in China

**DOI:** 10.7150/ijms.60780

**Published:** 2021-06-22

**Authors:** Jia-ying Wei, Zhou Xu, Hao Li, Wen-qin Du, Bai-ling Niu, Shu Li, Shen Tian, Juan Wu, Yu-ling Chen, Xin Li, Zi-li Liu, Jun Xiao, Guo-sheng Ren, Liang Ran, Ling-quan Kong

**Affiliations:** 1 Department of Endocrine and Breast Surgery, The First Affiliated Hospital of Chongqing Medical University, Chongqing 400016, China.; 2 Department of Thyroid and Breast Surgery, Affiliated Hospital of North Sichuan Medical College, Nanchong, Sichuan 637000, China.; 3 Department of Statistics, Colorado State University, Fort Collins, Colorado 80523, USA.; 4 Department of Intensive Care Medicine, The First Affiliated Hospital of Chongqing Medical University, Chongqing 400016, China.; 5 The Health Management Center of the First Affiliated Hospital of Chongqing Medical University, Chongqing 400016, China.

**Keywords:** Metabolic associated fatty liver disease, body mass index, endometrial thickness, postmenopausal women, ultrasonography

## Abstract

**Objectives:** To determine the relationship between the endometrial thickness (ET) and metabolic associated fatty liver disease (MAFLD) in the postmenopausal women who have a comprehensive health examination.

**Methods:** This was a population-based, retrospective observational study of the prevalence of MAFLD in 8594 postmenopausal women with different ET in the Quality Control Center of Health Examination in Chongqing, China. Binary and multivariable logistic regression analyses were used to obtain odds ratios and 95% confidence intervals for patients of different ET with MAFLD after adjusting for age.

**Results:** The incidences of MAFLD were 28.6% (1352), 30.3% (1058), 34.9% (133) in postmenopausal women with ET of < 3 mm, 3 mm ≤ & < 5 mm, and ≥ 5 mm, respectively. Compared with a baseline ET of less than 5.0 mm, the risk of MAFLD in patients with ET of ≥5.0 mm is higher (OR=1.291, 95% CI: 1.041-1.603, P<0.05). After adjustment for age, a statistically significant positive correlation was still observed. The increased prevalence of MAFLD in patients with ET of 3 mm ≤ &<5 mm (OR=1.110, 95% CI: 1.008-1.223) and ≥5 mm (OR=1.383, 95% CI: 1.109-1.724) achieved statistical significance, respectively. In addition, multiple logistic analyses controlling for age also confirmed the finding of positive correlation among body mass index (BMI) and ET.

**Conclusion:** Our results suggest that there is a positive correlation between MAFLD and ET in postmenopausal women. In addition, increased BMI is also associated with an increased risk of thickened endometrium.

## Introduction

Nonalcoholic fatty liver disease (NAFLD), defined as the fat infiltration into liver, is now recognized as one of the most common causes of chronic liver disease in the developed world 1. It is estimated that the global prevalence rate of NAFLD in the general population is 24%-25.2%, which may put more pressure on the healthcare system 2. Also, NAFLD is a type of metabolic stress-induced liver damage closely related to metabolic syndrome. Recently, the concept of metabolic associated fatty liver disease (MAFLD) has been proposed to provide a better understanding of potential liver dysfunction in metabolic diseases by an international expert consensus statement 3-6. The proposed MAFLD can eliminate the potential impact of other chronic liver diseases and excessive drinking on the diagnosis 7-11. In addition, to determine fatty liver disease (FLD) prevalence in the general population, ultrasound evaluation is also the most widely used first-line diagnostic modality 12. As it is relatively inexpensive and has good estimated sensitivity (85%) and specificity (94%) for diagnosing moderate to severe steatosis 13. The Asian Pacific Association for the Study of the Liver (APASL) clinical practice guidelines recommended using abdominal ultrasound, a non-invasive tool, for the diagnosis of MAFLD 14.

Endometrial carcinoma is one of the most common gynecological cancers in women 15 and is closely related to hormone levels 1,16,17. Its incidence rate and mortality are increasing each year all over the world, especially in industrialized countries, which are estimated at about 76000 death in 2019 18. Depending on the American College of Obstetrics and Gynecology (ACOG) 2018 19, ultrasonography is a general and sensitive detection method of endometrial cancer. A review of approximately 2,900 postmenopausal women demonstrated that the sensitivity and specificity of ultrasound diagnosis of endometrial thickness (ET) of 5 mm are 90% and 54%, respectively for the detection of endometrial cancer 12. Postmenopausal women who underwent annual pelvic ultrasound screening are the most important detection method for endometrial cancer. ET over 5 mm was most likely to be defined as endometrial thickening 20-24 and study shows that the risk of endometrial cancer and endometrial hyperplasia with atypia in asymptomatic postmenopausal women is increased with ET ≥ 11 mm 25. ET of postmenopausal women is related to endometrial disease-related carcinoma 26,27.

Clinical researches verified the progression of FLD in advanced fibrosis 28, hepatocellular carcinoma 29 and even extrahepatic malignancy include endometrial cancer 30. Two recent studies have demonstrated that women with endometrial cancer have a significantly increased risk of NAFLD 1,31. That is to say, they may have the same underlying molecular mechanisms 1. However, there is actually no research on MAFLD 32 and ET in postmenopausal women, and the relationship between these two parameters is still unclear.

In China, asymptomatic women undergoing regular health check-ups usually chose to accept both abdominal and pelvic ultrasonography 17. So, we can do further investigations. The purpose of this study was to investigate the relationship between MAFLD and ET in the asymptomatic postmenopausal women.

## Material and methods

### Population

We obtained data from the Public Health Center of the First Affiliated Hospital of Chongqing Medical University (a large tertiary hospital in southwest China) which contains comprehensive information of the female population who underwent comprehensive health examination, including age, height, BMI, waist circumference, metabolic components, and abdominal and gynecological ultrasonography imaging findings. This was a population-based, retrospective observational study of the prevalence of MAFLD investigated in consecutive postmenopausal women who underwent comprehensive health examination and accepted both abdominal and gynecological ultrasonography evaluation in the Quality Control Center of Health Examination in Chongqing, China, from January 2015 to July 2018. The characteristics of all examinees presenting with different ET were analyzed.

The ET of all participants was accurately determined by a 4-8 MHz vaginal transducer (HD11XE; Philips Medical Systems) or a 4-11 MHz transducer (Aplio 500; Canon Medical Systems), and then was routinely evaluated and recorded 33. As transvaginal sonography, the bladder was emptied at first, patients were examined in a lithotomy position scanned with an intravaginal ultrasound probe. The upper abdomen transabdominal ultrasound was scanned in the supine position with bladder filling. Scans were performed by sonographers with the same level of knowledge all came from the Department of Ultrasonography of the First Affiliated Hospital of Chongqing Medical University. All ultrasound scans were saved in the hard drive of the machine in digital imaging and communications in medicine format.

The data were collected and registered in the electronic medical record system of the Quality Control Center of Health Examination in Chongqing 33. Basic information such as age, gender, disease history, and postoperative ultrasound description was included. Anthropometric parameters such as blood pressure, weight, height, and waist circumference were measured by allied health professionals according to standard methods. Moreover, the blood samples of metabolic components were collected after fasting for at least 8 hours and analyzed in the laboratory of the First Affiliated Hospital of Chongqing Medical University certified by the College of American Pathologists (CAP Number: 7215494) 33. The study was approved and supervised by the Ethics Committee of The First Affiliated Hospital of Chongqing Medical University.

### Inclusion and Exclusion Criteria

We included 50897 women who underwent comprehensive health examination in the Quality Control Center of Health Examination in Chongqing, China, from January 2015 to July 2018(the health examiners are consecutive). Then excluded those who had a hysterectomy, cholecystectomy 34-36, ovariectomy 37,38 or a history of relative malignancy, intrauterine device *in situ* or if they were during pregnant, puerperium periods or breast feeding 39. Also, the cases without the original descriptions and detailed reports of abdominal and gynecological ultrasonography (include ET) or without available data of age, weight, height, body mass index, or correlated metabolic components 40 were ruled out. Finally, 8594 postmenopausal women were enrolled in this study.

### Statistical analysis

The postmenopausal women were divided into three groups: (1) the postmenopausal women with ET of <3 mm (2) the postmenopausal women with ET of 3 mm ≤ & < 5 mm and (3) the postmenopausal women with ET of ≥ 5 mm (the cutoff threshold for ET has been used in previous studies 20-24). Statistical analysis was performed with Microsoft Excel 2013, SPSS 23.0, and R software in this study. P value of <0.05 was considered to be statistically significant. All data were analyzed according to the intention-to-treat principle. Mean [std. deviation] and number (percentage) were used to describe demographic and clinical data. Kruskal Wallis test and Chi-square test were used to compare subject characteristics and determine consistency. Multivariable and binary logistic regression analyses were used to obtain odds ratios and 95% confidence intervals for patients of different ET with FLD after adjusting for age.

## Results

**Figure 1:**
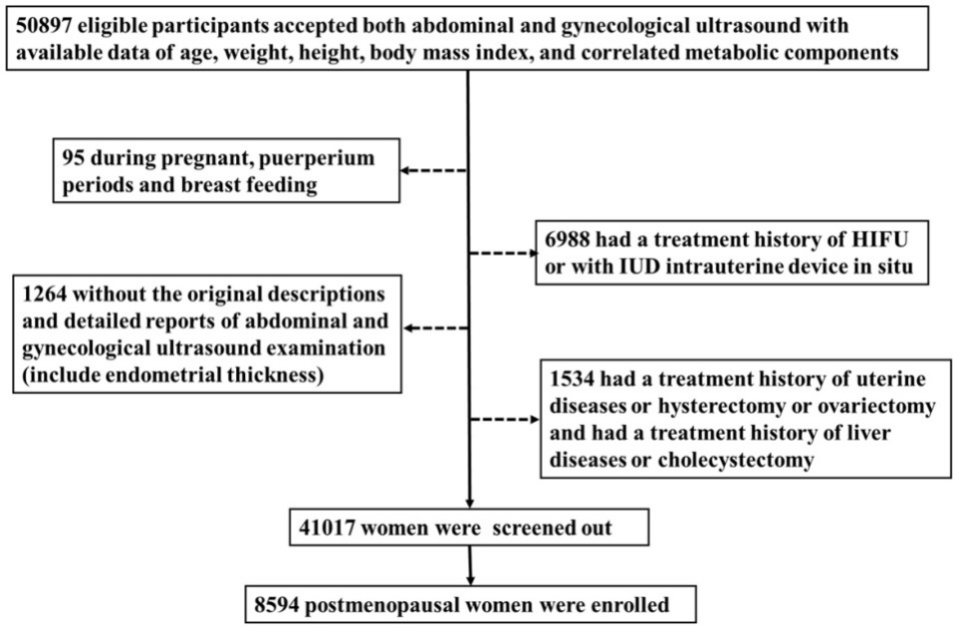
** Flow chart of study participants.** A total of 50897 women participated in this study of whom 8594 were postmenopausal. Among the postmenopausal women, 381 (4.4%) had an ET greater than or equal to 5 mm, of whom 137 (36.0%) presented with fatty liver based on transabdominal ultrasonography and 133 (34.9%) presented with MAFLD.

**Figure 2:**
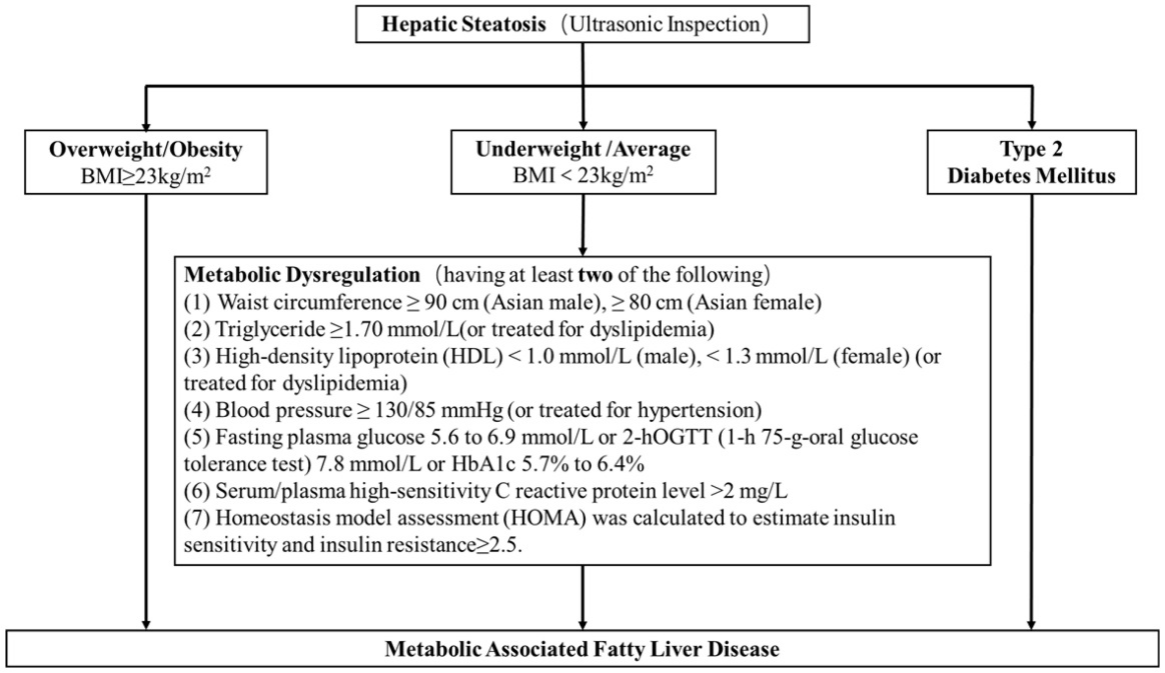
** The diagnostic criteria of MAFLD.** The diagnostic criteria of MAFLD are based on evidence of hepatic steatosis, one of the following three criteria, namely overweight/obesity, presence of type 2 diabetes mellitus, or evidence of metabolic dysregulation3 which was defined as having at least two of the following; (1) waist circumference ≥ 90 cm (Asian male), ≥ 80 cm (Asian female), (2) triglyceride ≥1.70 mmol/L (or treated for dyslipidemia), (3) high-density lipoprotein (HDL) < 1.0 mmol/L (male), < 1.3 mmol/L (female) (or treated for dyslipidemia), (4) blood pressure ≥ 130/85 mmHg (or treated for hypertension), (5) fasting plasma glucose 5.6 to 6.9 mmol/L or 2-h OGTT (1-h 75-g-oral glucose tolerance test (OGTT) 7.8 mmol/L or HbA1c 5.7% to 6.4%, (6) serum/plasma high-sensitivity C reactive protein level >2 mg/L, (7) homeostasis model assessment (HOMA) 2 was calculated to estimate insulin sensitivity and insulin resistance ≥2.5.

**Figure 3:**
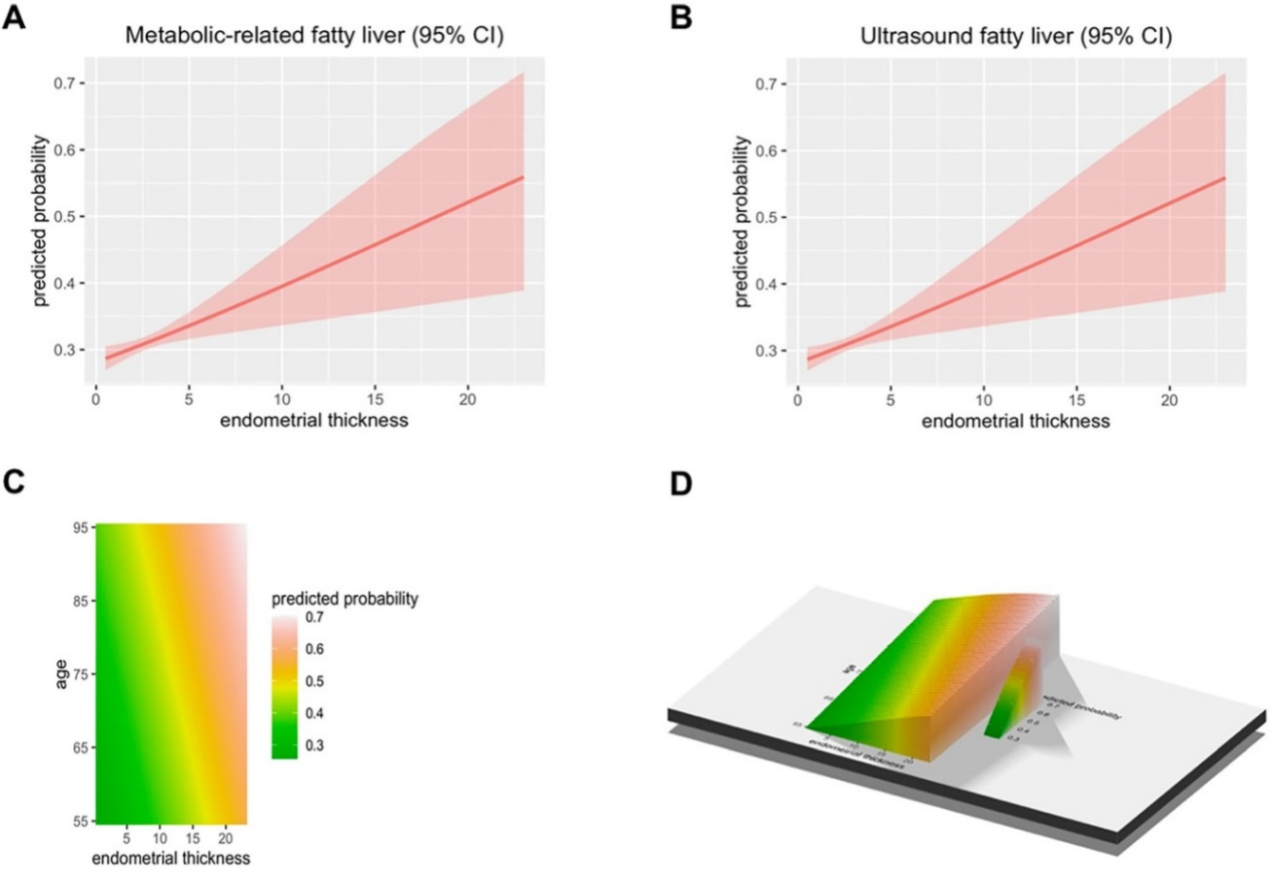
** The predicted probability of metabolic associated fatty liver disease (MAFLD) and ultrasound based fatty liver disease.** (A) The Endometrial thickness predicting the presence of Metabolic associated fatty liver disease were examined by the R. (B) The Endometrial thickness predicting the presence of Ultrasonic fatty liver were examined by the R. (C-D) The Endometrial thickness and age predicting the presence of FLD were examined by the R.

A total of 50897 women participated in this study of whom 8594 were postmenopausal (Figure 1). Among the postmenopausal women, 381 (4.4%) had an ET greater than or equal to 5 mm, of whom 137 (36.0%) presented with fatty liver based on transabdominal ultrasonography and 133 (34.9%) presented with MAFLD. The diagnostic criteria of MAFLD are based on evidence of hepatic steatosis (Figure 2), in addition to one of the following three criteria, namely overweight/obesity, presence of type 2 diabetes mellitus, or evidence of metabolic dysregulation 3 which was defined as having at least two of the following; (1) waist circumference ≥ 90 cm (Asian male), ≥ 80 cm (Asian female), (2) triglyceride ≥1.70 mmol/L (or treated for dyslipidemia), (3) high-density lipoprotein (HDL) < 1.0 mmol/L (male), < 1.3 mmol/L (female) (or treated for dyslipidemia), (4) blood pressure ≥ 130/85 mmHg (or treated for hypertension), (5) fasting plasma glucose 5.6 to 6.9 mmol/L or 2-h OGTT (1-h 75-g-oral glucose tolerance test (OGTT) 7.8 mmol/L or HbA1c 5.7% to 6.4%, (6) serum/plasma high-sensitivity C reactive protein level >2 mg/L, (7) homeostasis model assessment (HOMA) 2 was calculated to estimate insulin sensitivity and insulin resistance ≥2.5. The ET (and age) predicting the presence of FLD were examined by the R (Figure 3).

The baseline characteristics of all participants with different ET are shown in Table 1. The postmenopausal women with ET of ≥ 5 mm were more likely to be overweight or obese, as well as have elevated waist circumference, blood pressure, triglycerides, fasting blood sugar, and reduced high-density lipoprotein cholesterol.

The results of women with abdominal and gynecological ultrasound imaging findings are shown in Table 2. If the expected frequency counts count less than 5, the chi-square test was conducted. There was a weak positive correlation between the prevalence of ultrasonic based fatty liver disease and different endometrial thickness. The prevalence of ultrasonic based fatty liver disease were 30.0% (1417), 31.7% (1109), 36.0% (137) in postmenopausal women with ET of less than 3 mm, 3 mm ≤ & <5 mm, and ≥5 mm, respectively. While the incidences of MAFLD were 28.6% (1352), 30.3% (1058), 34.9% (133) in postmenopausal women with ET of <3 mm, 3 mm ≤ & <5 mm, and ≥5 mm, respectively.

To evaluate the correlation between the FLD and ET (Table 3), the number of the postmenopausal women with endometrial thickness of less than 3 mm is 8213, and the number of postmenopausal women with endometrial thickness of greater than or equal to 5 mm is 381. If the 95% confidence interval from the risk estimate did not include 1, the odds ratio was considered significant. When a postmenopausal woman has an ET of greater than or equal to 5 mm, the risk of ultrasonic fatty liver is higher (OR=1.264, 95% CI: 1.020-1.566) and the risk of MAFLD is higher too (OR=1.291, 95% CI: 1.041-1.603).

Our study used binary logistic regression analysis (Table 4). The unadjusted one finds that compared with a baseline ET of less than 3.0 mm, the increased prevalence of the ultrasonic based fatty liver disease in patients with 3mm≤ET<5 mm (OR=1.084, 95% CI: 0.986-1.192) and MAFLD (OR=1.082, 95% CI: 0.983-1.191) is not statistically significant. While the increased prevalence of the ultrasonic based fatty liver disease in patients with ET≥5 mm (OR=1.309, 95% CI: 1.052-1.628) and MAFLD (OR=1.336, 95% CI: 1.072-1.665) are both statistically significant. After adjustment for age, a statistically significant positive correlation was observed. The increased prevalence of the ultrasonic based fatty liver disease in patients with 3mm≤ET<5 mm (OR=1.107, 95% CI: 1.007-1.218) and MAFLD (OR=1.110, 95% CI: 1.008-1.223) achieved statistical significance; and the increased prevalence of the ultrasonic based fatty liver disease in patients with ET ≥5 mm (OR=1.347, 95% CI: 1.082-1.676) and MAFLD (OR=1.383, 95% CI: 1.109-1.724) also achieved statistical significance. In addition, hazard ratios of ultrasonic based fatty liver disease (OR=1.052, 95% CI: 1.017-1.089) and MAFLD (OR=1.056, 95% CI: 1.021-1.093) were standardized by calculating them for 1 mm increment of each continuous variable endometrial thickness. Another age-adjusted one shows that the risk of ultrasonic based fatty liver disease and MAFLD are1.060 and 1.065 times for 1 mm increment of ET.

Multiple logistic analyses controlling for age confirmed the finding that a significant positive correlation was detected among BMI and ET (Table 5). Compared with a baseline ET of less than 3.0 mm, the increased prevalence of 3mm≤ET<5 mm achieved statistical significance by overweight BMI≥23~24.9 (OR=1.795, 95% CI: 1.005-3.203), obese I BMI≥25~29.9 (OR=2.458, 95% CI: 1.388-4.354), obese II BMI≥30 (OR=3.409, 95% CI: 1.343-8.654). While ET≥5 mm is also statistically significant by obese I BMI≥25~29.9 (OR=1.821, 95% CI: 1.024-3.238), obese II BMI≥30 (OR=2.562, 95% CI: 1.002-6.550).

## Discussion

Obesity is a very serious public health problem around the world and an important contributing factor for the metabolic syndrome, which is an established risk factor associated with cancer risk and mortality 41, including endometrial cancer 42 and fatty liver disease-related hepatocellular carcinoma 43. Relevant research found that obesity-related cancer may be related to MAPK signalling 41. World Health Organization (WHO) reports recently that about 2 billion (39%) adults are overweight and over 600 million (13%) are obese. Another study performed from 1975 to 2014 in 19.2 million adult participants, reported that the age-standardized prevalence of obesity increased from 6.4% in 1975 to 14.9% in 2014 in women and both were higher than male 29. In addition, women may be more susceptible than men to the effects of estrogen.

Obesity and estrogen 44 are the strong risk factor for endometrial cancer 42,45. Also, obesity has been found to increase endometrial thickness independently 46-48 and other pathologies in post-menopausal women through an action on androgen concentrations 49,50. Here, the ET analysis was used to predict the occurrence of endometrial disease and BMI to represent the degree of overweight and obesity. The clinical significance of ET can be applied to investigate endometrial abnormalities in asymptomatic women 51 and can lie in the early detection of endometrial carcinoma 52. There was evidence that the prevalence of FLD generally increased incrementally with greater BMI 13. Increased endometrial thickness is associated with estrogen levels 33. Studies have been reported that there was a positive and significant correlation between BMI of ≥30 kg/m^2^ and ET, demonstrating the influence of obesity on endometrial thickening 53,54. Two recent studies have demonstrated that surgical menopause results in a significantly increased risk of FLD in women with endometrial cancer 1,31. Also, treating obesity by bariatric surgery can improve endometrial cancer survivorship 42.

The results of this study indicated a significant correlation between MAFLD and ET without adjusting for any other risk factors using the whole sample. There is a positive correlation between MAFLD and ET in the general population. This significant correlation persisted after adjustment for age. So, we have reason to believe that this study supports that there is a statistical correlation between MAFLD or ultrasonic fatty liver and ET. In addition, we speculate that the ET is closely related to obesity and abdominal fat distribution. It is noteworthy to exclude BMI to minimize this as a confounding factor. However, after adjusting for age, multiple logistic did confirm the findings that a significant positive correlation was detected among BMI and ET. This is an important finding of our study and it also highlights the impact of obesity on ET.

The study has several limitations. Most importantly, the limited number of female cheek-up crowd postmenopausal women with imaging examination and metabolic components only restricted conclusions concerning the significance, especially when divided into subgroups. Second, the retrospective study design does not permit to fully reproduction the reasons for an indication of surgical excision like abortion operation in all cases. However, further longitudinal research with larger participants cohorts or meta-analyses of existing surveys is necessary to explore the relationship between MAFLD and the ET.

If postmenopausal women, especially obese people, have developed MAFLD, then a pelvic ultrasound needed to be performed to monitor the ET for detecting early-stage endometrial cancer. Our study also demonstrated the value of controlling obesity and promoting healthy weight in the population. It is therefore an optimal key to improve quality of life and prevent the occurrence of fatty liver disease and gynecological disease worldwide by healthy weight control behaviors. Interestingly, histological improvement in liver biopsies is associated with the extent of weight loss 29. Unfortunately, to date, none of the pharmacological approaches have provided a real, long-lasting benefit. With the obvious exception of genetics, almost all of the susceptibility factors for fatty liver disease are theoretically modifiable, highlighting the potential impact of education and policy in attenuating the burden of it 55,56. Thus, the cornerstones to the management of this disease are still lifestyle modifications and weight loss 57.

## Supplementary Material

Supplementary figures and tables.

## Figures and Tables

**Table 1:** Characteristics of women included in the study (n=8594) by endometrial thickness^a^

	Endometrial thickness (n=8594)		
	<5 mm (n=8213)	≥5 mm (n=381)	*P* value^b^
***Baseline characteristic***			
Age, y	63. 55 [6.70]	62.30 [6.76]	<0.001
Height, cm	154.27[5.64]	155.21 [6.06]	0.005
**Body mass index^c^**			
BMI	23.55 [3.03]	24.34 [3.05]	<0.001
Underweight <18.5	272(3.3%)	0 (0.0%)	0.001
Average18.5~22.9	3459 (42.1%)	481 (18.1%)	
Overweight ≥23~24.9	2080 (25.3%)	738 (27.7%)	
Obese I ≥25~29.9	2194 (26.7%)	1265 (47.5%)	
Obese II ≥30	208 (2.5%)	179 (6.7%)	
***Metabolic components^d^***			
**Waist circumference**			
Waist, cm	79.85 [7.89]	81.74 [7.85]	<0.001
Normal	3982 (48.5%)	135 (35.4%)	<0.001
Elevated ≥ 80 cm	4231 (51.5%)	246 (64.6%)	
**Blood pressure**			
SBP, mmHg	133.25 [19.86]	135.07 [20.68]	0.091
DBP, mmHg	76.48 [11.05]	77.40 [12.34]	0.249
Normal	3603 (43.9%)	156 (40.9%)	0.261
Elevated ≥130/85 mmHg	4610 (56.1%)	225 (59.1%)	
**Triglycerides**			
Triglycerides, mmol/L	1.64 [1.15]	1.60 [0.80]	0.671
Normal	5484 (66.8%)	247 (64.8%)	0.432
Elevated ≥1.7 mmol/L	2729 (33.2%)	134 (35.2%)	
**HDL**			
HDL-C, mmol/L	1.61 [0.37]	1.56 [0.34]	0.017
Normal	6630 (80.7%)	289 (75.9%)	0.019
Reduced <1.3 mmol/L	1583 (19.3%)	92 (24.1%)	
**Fasting blood sugar**			
Fasting glucose, mmol/L	5.76 [1.35]	5.81 [1.34]	0.522
Normal <5.6 mmol/L	4625 (56.3%)	205 (53.8%)	0.492
Elevated 5.6~6.9 mmol/L	2849 (34.7%)	136 (35.7%)	
Type 2 diabetes >6.9 mmol/L	739 (9.0%)	40 (10.5%)	

a. Values are given as mean [std. deviation] and number (percentage). b. Some P values were compared using Kruskal-Wallis tests. Other variables were derived from χ2 tests. c. Cut-off values of body mass index were defined as follows: underweight <18.5; average18.5~22.9; overweight ≥23~24.9; obese I ≥25~29.9; obese II ≥30. d. Cut-off values of metabolic components were defined as follows: waist circumference ≥80 cm; systolic blood pressure ≥130 mm Hg or diastolic blood pressure ≥85 mm Hg; triglycerides ≥1.7 mmol/L; HDL cholesterol < 1.3 mmol/L; fasting glucose from 5.6 to 6.9 mmol/L.

**Table 2:** The risk of metabolic associated fatty liver disease (MAFLD) and ultrasound-based fatty liver disease in relation to endometrial thickness^a^

Endometrial thickness/mm	Liver status				
	Total (n=8594)	Model 1		Model 2	
		Ultrasonic fatty liver^b^ (-) (n=5931)	Ultrasonic fatty liver (+) (n=2663)	MAFLD^c^ (-) (n=6051)	MAFLD (+) (n=2543)
<3	4720 (54.9%)	3303 (70.0%)	**1417 (30.0%)**	3368(71.4%)	**1352 (28.6%)**
3≤ET<5	3493 (40.6%)	2384 (68.3%)	**1109 (31.7%)**	2435(69.7%)	**1058 (30.3%)**
≥5	381 (4.4%)	244 (64.0%)	**137 (36.0%)**	248(65.1%)	**133 (34.9%)**

a. Data are given as a number (%). b. Ultrasonic fatty liver means fatty liver disease diagnosed by abdominal or gynecological ultrasonography. c. MAFLD means metabolic associated with fatty liver disease.

**Table 3:** The odds ratio for metabolic associated fatty liver disease (MAFLD) and ultrasound-based fatty liver disease in relation to endometrial thickness

Endometrial thickness/mm	Liver status				
	Total (n=8594)	Model 1		Model 2	
		Ultrasonic fatty liver (+) (n=2663)		MAFLD (+) (n=2543)	
	Number (%)	OR (95%CI)^a^	P value	OR (95%CI)^b^	P value
<5	8213 (95.6%)	1 (Reference)	0.032	1 (Reference)	0.020
≥5	381 (4.4%)	**1.264 (1.020 -1.566)**		**1.291 (1.041-1.603)**	

a. OR: odds ratio; 95%CI: 95% confidence interval. b. The odds ratio of fatty liver disease is adjusting for nothing.

**Table 4:** Binary logistic regression analysis for metabolic associated fatty liver disease (MAFLD) and ultrasound-based fatty liver disease in relation to endometrial thickness

	Liver status			
	Ultrasonic fatty liver^a^ (+) (n=2663)		MAFLD (+) (n=2543)	
	OR (95%CI)^ b^	P_trend_	OR (95%CI)	P_trend_
**Binary model 1**				
Endometrial thickness, mm	**1.052 (1.017-1.089)**	0.003	**1.056 (1.021-1.093)**	0.002
ET<3 mm	1 (Reference)	0.025	1 (Reference)	0.018
3 mm ≤ ET < 5 mm	1.084 (0.986-1.192)	0.093	1.082 (0.983-1.191)	0.106
ET ≥ 5mm	**1.309 (1.052-1.628)**	0.016	**1.336 (1.072-1.665)**	0.010
**Binary model 2**				
Age^c^, y	1.015 (1.008-1.022)	<0.001	1.018 (1.012-1.025)	<0.001
Endometrial thickness, mm	**1.060 (1.024-1.096)**	0.001	**1.065 (1.029-1.102)**	<0.001
ET <3 mm	1 (Reference)	0.008	1 (Reference)	0.004
3 mm ≤ET< 5 mm	**1.107 (1.007-1.218)**	0.036	**1.110 (1.008-1.223)**	0.034
ET ≥ 5 mm	**1.347 (1.082-1.676)**	0.008	**1.383 (1.109-1.724)**	0.004

a. Ultrasonic fatty liver means fatty liver disease diagnosed by abdominal or gynecological ultrasonography. b. The odds ratio of fatty liver disease is adjusting for age. c. In models, age and endometrial thickness were treated as a continuous variable.

**Table 5:** Multivariable adjusted analysis for BMI in relation to endometrial thickness

	Endometrial thickness, mm^b^			
	3mm ≤ ET < 5 mm		ET ≥ 5 mm	
	OR (95%CI)	P_trend_	OR (95%CI)	P_trend_
Age, y	1.050 (1.032-1.068)	< 0.001	1.016 (0.998-1.035)	0.073
**Body mass index^a^**				
Average, 18.5≤BMI≤22.9	1.337 (0.756-2.366)	0.318	1.192 (0.671-2.118)	0.549
Overweight, BMI≥23~24.9	**1.795 (1.005-3.203)**	**0.048**	1.430 (0.798-2.563)	0.230
Obese I, BMI≥25~29.9	**2.458 (1.388-4.354)**	**0.002**	**1.821 (1.024-3.238)**	**0.041**
Obese II, BMI≥30	**3.409 (1.343-8.654)**	**0.010**	**2.562 (1.002-6.550)**	**0.050**

a. The body mass index is divided into 5 groups according to Asian BMI classification. b. Underweight BMI<18.5: 1 (Reference).
