# Supplementary material for: Relationship between the Metabolic Associated Fatty Liver Disease and Endometrial Thickness in Postmenopausal Women: A Cross-sectional Study in China
Source: Int J Med Sci. 2021 Jun 22;18(14):3082–9. doi: 10.7150/ijms.60780 (PMC8364468; doi:10.7150/ijms.60780)
Supplement: Supplementary file 1 — Supplementary figures and tables. [file ijmsv18p3082s1.pdf]

Relationship between the metabolic associated fatty liver disease and endometrial thickness in postmenopausal women: a cross-sectional study in China

Supplementary material

Figures and Tables:

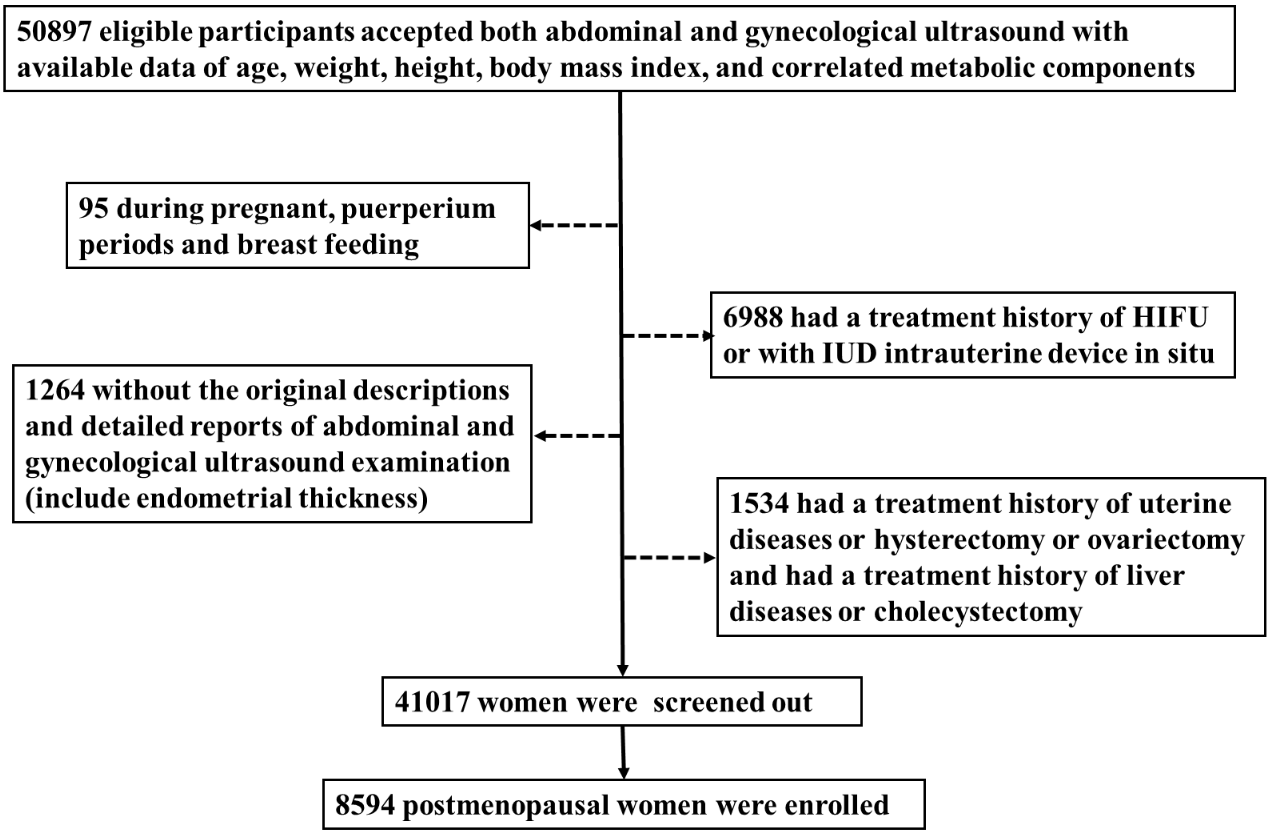

Figure 1 Flow chart of study participants.

Figure 1: A total of 50897 women participated in this study of whom 8594 were postmenopausal. Among the postmenopausal women,381(4.4%) had an ET greater than or equal to 5 mm, of whom 137 (36.0%) presented with fatty liver based on transabdominal ultrasonography and 133 (34.9%) presented with MAFLD.

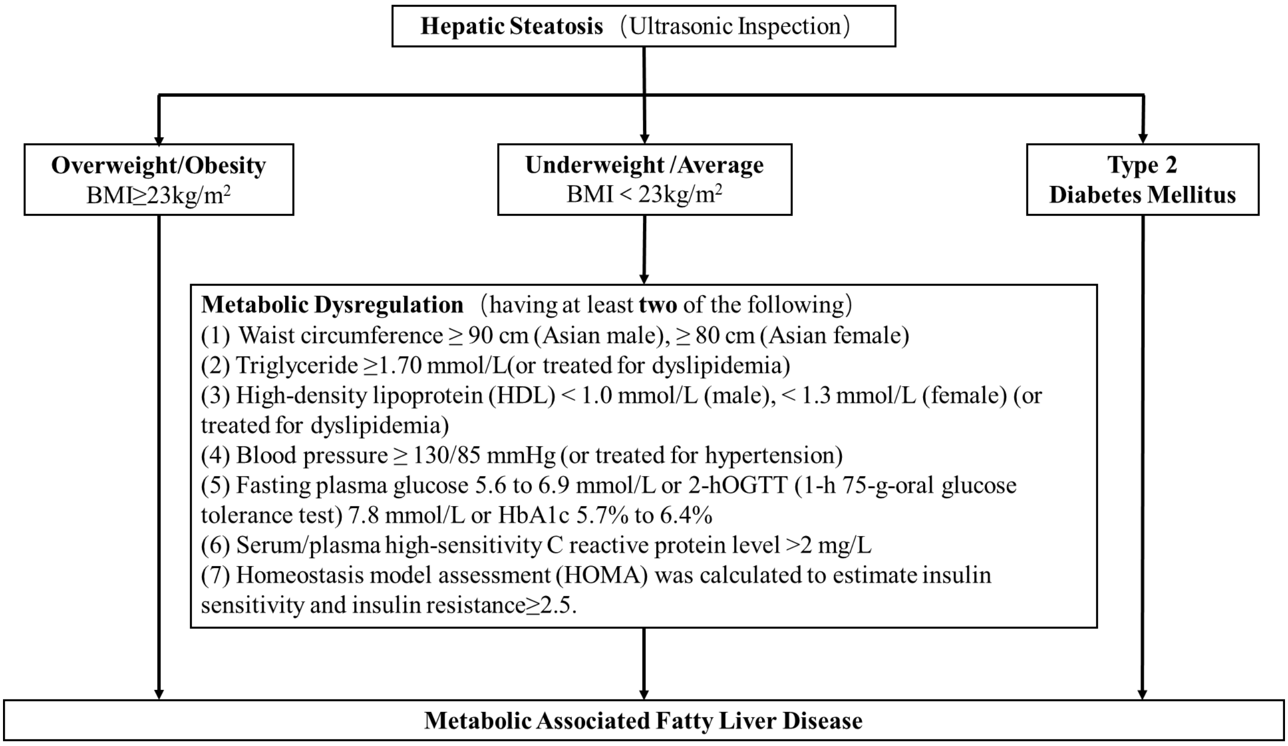

Figure 2 The diagnostic criteria of MAFLD.

Figure 2: The diagnostic criteria of MAFLD are based on evidence of hepatic steatosis, one of the following three criteria, namely overweight/obesity, presence of type 2 diabetes mellitus, or evidence of metabolic dysregulation3 which was defined as having at least two of the following; (1) waist circumference ≥ 90 cm (Asian male), ≥ 80 cm (Asian female), (2) triglyceride ≥1.70 mmol/L(or treated for dyslipidemia), (3) high-density lipoprotein (HDL) < 1.0 mmol/L (male), < 1.3 mmol/L (female) (or treated for dyslipidemia), (4) blood pressure ≥ 130/85 mmHg (or treated for hypertension), (5) fasting plasma glucose 5.6 to 6.9 mmol/L or 2-h OGTT (1-h 75-g-oral glucose tolerance test (OGTT) 7.8 mmol/L or HbA1c 5.7% to 6.4%, (6) serum/plasma high-sensitivity C reactive protein level >2 mg/L, (7) homeostasis model assessment (HOMA) 2 was calculated to estimate insulin sensitivity and insulin resistance≥2.5.

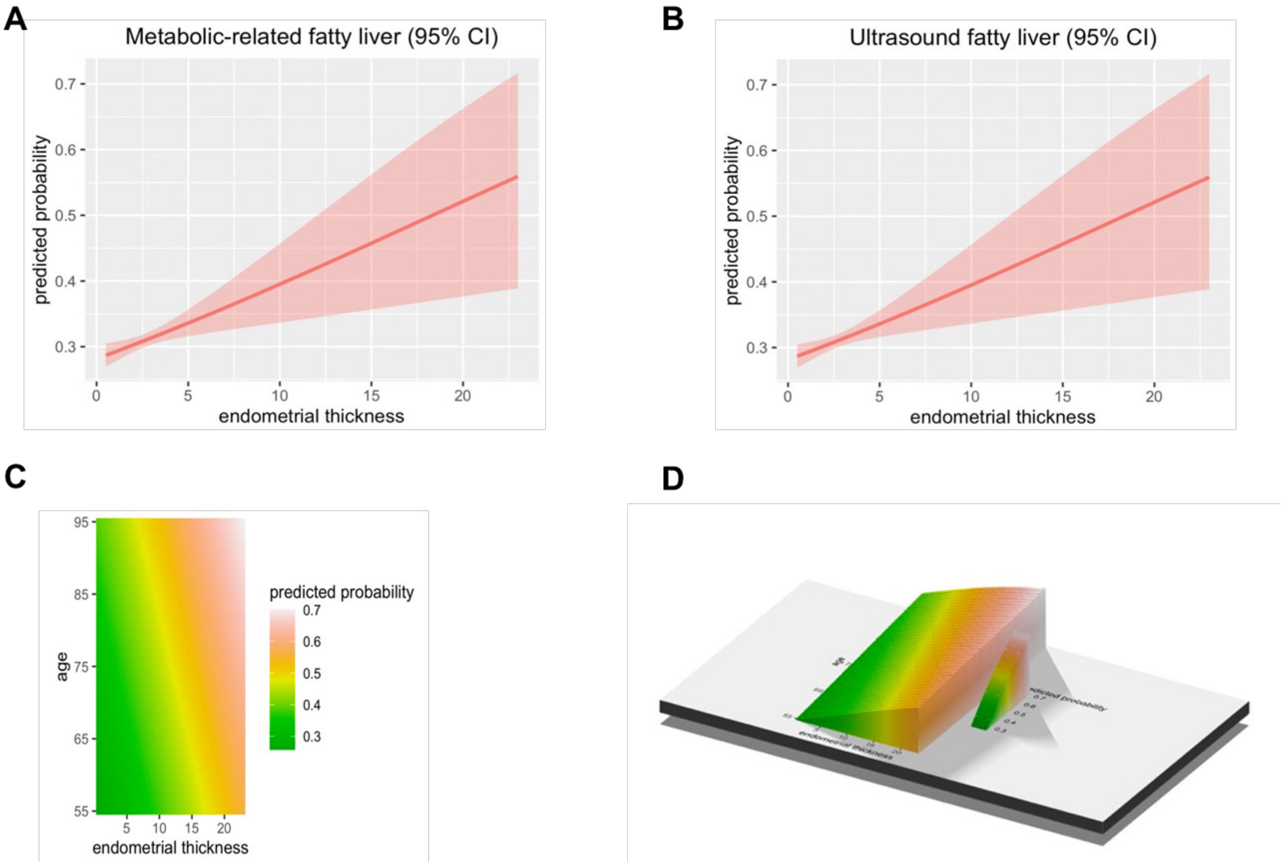

Figure 3　The predicted probability of metabolic associated fatty liver disease (MAFLD) and ultrasound based fatty liver disease.

Figure 3: (A) The Endometrial thickness predicting the presence of Metabolic associated fatty liver disease were examined by the R.(B) The Endometrial thickness predicting the presence of Ultrasonic fatty liver were examined by the R.(C-D) The Endometrial thickness and age predicting the presence of FLD were examined by the R.

Table 1 Characteristics of women included in the study (n=8594) by endometrial thickness. [a]

	Endometrial thickness (n=8594)		
	＜5mm（n=8213）	≥5mm（n=381）	*P* value [b]
**Baseline characteristic**			
**Age, y**	63. 55 [6.70]	62.30[6.76]	＜0.001
**Height, cm**	154.27[5.64]	155.21 [6.06]	0.005
**Body mass index** [c]			
**BMI**	23.55 [3.03]	24.34[3.05]	＜0.001
**Underweight＜18.5**	272(3.3%)	0(0.0%)	0.001
**Average18.5~22.9**	3459 (42.1%)	481(18.1%)	
**Overweight≥23~24.9**	2080 (25.3%)	738(27.7%)	
**Obese I ≥25~29.9**	2194(26.7%)	1265(47.5%)	
**Obese II ≥30**	208(2.5%)	179(6.7%)	
**Metabolic components** [d]			
**Waist circumference**			
**Waist, cm**	79.85 [7.89]	81.74[7.85]	＜0.001
**Normal**	3982(48.5%)	135(35.4%)	＜0.001
**Elevated≥80cm**	4231(51.5%)	246(64.6%)	
**Blood pressure**			
**SBP, mmHg**	133.25[19.86]	135.07[20.68]	0.091
**DBP, mmHg**	76.48[11.05]	77.40[12.34]	0.249
**Normal**	3603 (43.9%)	156(40.9%)	0.261
**Elevated≥130/85 mmHg**	4610 (56.1%)	225(59.1%)	
**Triglycerides**			
**Triglycerides, mmol/L**	1.64[1.15]	1.60[0.80]	0.671
**Normal**	5484(66.8%)	247(64.8%)	0.432

**Elevated≥1.7 mmol/L**	2729(33.2%)	134(35.2%)	
**HDL**			
**HDL-C, mmol/L**	1.61[0.37]	1.56[0.34]	0.017
**Normal**	6630(80.7%)	289(75.9%)	0.019
**Reduced < 1.3 mmol/L**	1583(19.3%)	92(24.1%)	
**Fasting blood sugar**			
**Fasting glucose, mmol/L**	5.76[1.35]	5.81 [1.34]	0.522
**Normal < 5.6 mmol/L**	4625(56.3%)	205(53.8%)	0.492
**Elevated 5.6~6.9 mmol/L**	2849(34.7%)	136(35.7%)	
**Type 2 diabetes > 6.9 mmol/L**	739(9.0%)	40(10.5%)	

a. Values are given as mean [std. deviation] and number (percentage).

b. Some P values were compared using Kruskal-Wallis tests. Other variables were derived from $\chi2$ tests.

c. Cut-off values of body mass index were defined as follows: underweight < 18.5; average18.5~22.9; overweight≥23~24.9; obese I ≥25~29.9; obese II ≥30.

d. Cut-off values of metabolic components were defined as follows: waist circumference ≥80 cm; systolic blood pressure ≥130 mm Hg or diastolic blood pressure ≥85 mm Hg; triglycerides ≥1.7 mmol/L; HDL cholesterol < 1.3 mmol/L; fasting glucose from 5.6 to 6.9 mmol/L.

Table 2 The risk of metabolic associated fatty liver disease (MAFLD) and ultrasound-based fatty liver disease in relation to endometrial thickness. [a]

Endometrial thickness / mm	Liver status				
	Total	Model 1		Model 2	
	（n=8594）	Ultrasonic fatty liver[b] （-）（n=5931）	Ultrasonic fatty liver （+）（n=2663）	MAFLD[c]（-） （n=6051）	MAFLD（+） （n=2543）
**＜3**	4720(54.9%)	3303 (70.0%)	**1417 (30.0%)**	3368(71.4%)	**1352(28.6%)**
**3≤ET＜5**	3493(40.6%)	2384(68.3%)	**1109 (31.7%)**	2435(69.7%)	**1058(30.3%)**
**≥5**	381(4.4%)	244 (64.0%)	**137 (36.0%)**	248(65.1%)	**133(34.9%)**

a. Data are given as a number (%).

b. Ultrasonic fatty liver means fatty liver disease diagnosed by abdominal or gynecological ultrasonography.

c. MAFLD means metabolic associated with fatty liver disease.

Table 3 The odds ratio for metabolic associated fatty liver disease (MAFLD) and ultrasound-based fatty liver disease in relation to endometrial thickness.

Endometrial thickness / mm	Liver status				
	Total（n=8594）	Model 1		Model 2	
		Ultrasonic fatty liver（+）（n=2663）		MAFLD（+）（n=2543）	
	number (%)	OR (95%CI)[a]	P value	OR (95%CI)[b]	P value
**＜5**	8213(95.6%)	1 (Reference)	0.032	1 (Reference)	0.020
**≥5**	381(4.4%)	**1.264 (1.020 -1.566)**		**1.291(1.041-1.603)**	

a. OR: odds ratio; 95%CI: 95% confidence interval.

b. The odds ratio of fatty liver disease is adjusting for nothing.

Table 4 Binary logistic regression analysis for metabolic associated fatty liver disease (MAFLD) and ultrasound-based fatty liver disease in relation to endometrial thickness.

	Liver status			
	Ultrasonic fatty liver[a]（+）（n=2663）		MAFLD（+）（n=2543）	
	OR (95%CI) [b]	P_{trend}	OR (95%CI)	P_{trend}
**Binary model 1**				
**Endometrial thickness, mm**	**1.052(1.017-1.089)**	0.003	**1.056(1.021-1.093)**	0.002
**ET < 3 mm**	1 (Reference)	0.025	1 (Reference)	0.018
**3mm ≤ET < 5 mm**	1.084 (0.986-1.192)	0.093	1.082 (0.983-1.191)	0.106
**ET≥5mm**	**1.309 (1.052-1.628)**	0.016	**1.336 (1.072-1.665)**	0.010
**Binary model 2**				
**Age[c], y**	1.015(1.008-1.022)	< 0.001	1.018(1.012-1.025)	< 0.001
**Endometrial thickness, mm**	**1.060(1.024-1.096)**	0.001	**1.065(1.029-1.102)**	< 0.001
**ET < 3 mm**	1 (Reference)	0.008	1 (Reference)	0.004
**3mm ≤ET < 5 mm**	**1.107 (1.007-1.218)**	0.036	**1.110 (1.008-1.223)**	0.034
**ET≥5mm**	**1.347 (1.082 -1.676)**	0.008	**1.383 (1.109-1.724)**	0.004

a. Ultrasonic fatty liver means fatty liver disease diagnosed by abdominal or gynecological ultrasonography.

b. The odds ratio of fatty liver disease is adjusting for age.

c. In models, age and endometrial thickness were treated as a continuous variable.

Table 5 Multivariable adjusted analysis for BMI in relation to endometrial thickness.

	Endometrial thickness, mm[b]			
	3mm ≤ ET < 5 mm		ET ≥ 5 mm	
	OR (95%CI)	P_{trend}	OR (95%CI)	P_{trend}
**Age, y**	1.050(1.032-1.068)	< 0.001	1.016(0.998-1.035)	0.073
**Body mass index[a]**				
**Average 18.5≤BMI≤22.9**	1.337(0.756-2.366)	0.318	1.192(0.671-2.118)	0.549
**Overweight BMI≥23~24.9**	**1.795(1.005-3.203)**	**0.048**	1.430(0.798-2.563)	0.230
**Obese I BMI≥25~29.9**	**2.458(1.388-4.354)**	**0.002**	**1.821(1.024-3.238)**	**0.041**
**Obese II BMI≥30**	**3.409(1.343-8.654)**	**0.010**	**2.562(1.002-6.550)**	**0.050**

a. The body mass index is divided into 5 groups according to Asian BMI classification.

b. Underweight BMI < 18.5: 1 (Reference)
